# Supplementary material for: Ago1 Affects the Virulence of the Fungal Plant Pathogen Zymoseptoria tritici
Source: Genes (Basel). 2021 Jun 30;12(7):1011. doi: 10.3390/genes12071011 (PMC8303167; doi:10.3390/genes12071011)
Supplement: Supplementary file 1 [file genes-12-01011-s001.zip › genes-1264960 - supplementary.pdf]

Supplementary Materials

Table S1. Location of RNAi machinery homologs in IPO323

gene	gene ID*	chromosome	start	end
Ago1	Zt09_chr3_00833	chr3	2633932	2630540
Ago2	Zt09_chr11_00306	chr11	940658	943543
Ago3	Zt09_chr1_02012	chr1	5671029	5667619
Ago4	ZT09_chr1_01766	chr1	4993178	4996732
Dcl	ZT09_chr9_00498	chr9	1665453	166747

*[51]

Table S2. List of strains and assemblies used in this study

species	strain ID	Origin	reference
Z. tritici	IPO323	Netherlands	[53]
Z. tritici	ST00Arg	Argentina	[78]
Z. tritici	ST09TN	Tunisia	[78]
Z. tritici	ST10CRI	Czech Republic	[78]
Z. tritici	ST90Oreg	USA	[78]
Z. tritici	ST92ISY	Israel	[78]
Z. tritici	ST92YEQ	Yemen	[78]
Z. tritici	ST93CNR	Canada	[78]
Z. tritici	ST93I	USA	[78]
Z. tritici	ST94KE	Kenia	[78]
Z. tritici	ST95UR	Ukraine	[78]
Z. tritici	1A5	Switzerland	[79]
Z. tritici	1E4	Switzerland	[79]
Z. tritici	3D1	Switzerland	[79]
Z. tritici	3D7	Switzerland	[79]
Z. tritici	Zt02	Denmark	[51]
Z. tritici	Zt04	Denmark	[51]
Z. tritici	Zt05	Denmark	[51]
Z. tritici	Zt07	Denmark	[51]
Z. tritici	Zt148	Germany	[51]
Z. tritici	Zt150	Germany	[51]
Z. tritici	Zt151	Germany	[51]
Z. tritici	Zt152	Germany	[51]
Z. tritici	Zt154	Germany	[51]
Z. tritici	Zt155	Germany	[51]
Z. tritici	IPO94269	Netherlands	[41]
Z. tritici	Zt10	Iran	[66]
Z. tritici	Zt289	Iran	[80]
Z. tritici	Zt469	Iran	[80]
Z. ardabiliae	Za17	Iran	[81]
Z. ardabiliae	Za100	Iran	[81]
Z. brevis	Zb163	Iran	[51]
Z. brevis	Zb18110	Iran	[51]
Z. brevis	Zb87	Iran	[81]

Z. passerinii	Zpa63	USA	[81]
Z. pseudotritici	Zp13	Iran	[81]

Table S3. List of all primers used within this study.
see attached file

Table S4. Results of PCR-based karyotyping of evolved clones
see attached file

Table S5. Results of in planta phenotype characterisation
see attached file

Table S6: List of all strains included in the experimental evolution experiment and summary of results of experimental evolution experiment

Strain ID	Genotype	# technical replicates	# chromosomes not lost	# lost chromosomes
IPO323	wt	3	892	12
ZT117#91	Δ*Dcl*	3	433	3
ZT117#176	Δ*Dcl*	3	412	6
Zt194#1	Δ*Dcl::Dcl*	3	405	6
Zt194#22	Δ*Dcl::Dcl*	3	423	3
ZT108#42	Δ*Ago1*	3	555	1
ZT108#59a	Δ*Ago1*	3	851	6
Zt201#8	Δ*Ago1::Ago1_gfp*	3	298	1
Zt201#20	Δ*Ago1::Ago1_gfp*	3	413	5
Zt202#47	Δ*Ago2*	3	445	1
Zt202#61	Δ*Ago2*	3	394	3
Zt215#27	Δ*Ago2::Ago2_gfp*	3	314	2
Zt215#43	Δ*Ago2::Ago2_gfp*	3	423	3
Zt89#33	Δ*Ago3*	3	299	3
Zt89#40	Δ*Ago3*	3	444	3
Zt203#37	Δ*Ago3::Ago3_gfp*	3	424	4
Zt203#38	Δ*Ago3::Ago3_gfp*	3	451	2

Supplementary Figures

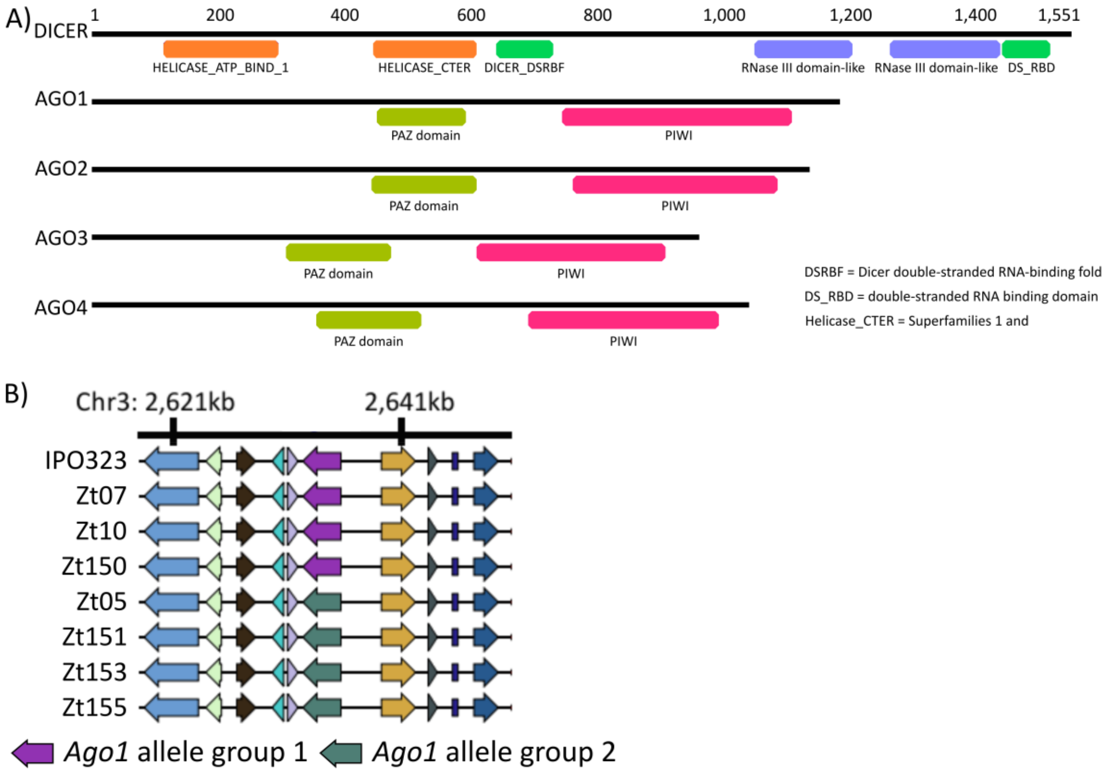

Figure S1. Protein domains and genomic localization of Argonaute and Dicer homologs in *Z. tritici.* A) Predicted Protein domains of Argonaute and Dicer protein homologs detected in Z. tritici. Domains were predicted using InterProScan. B) Genomic locations of representative examples of the two genetic groups of the Ago1 homologs in *Z. tritici*. Predicted genes (according to [49]) are depicted by filled arrows. The two genetic groups are located at the same locus

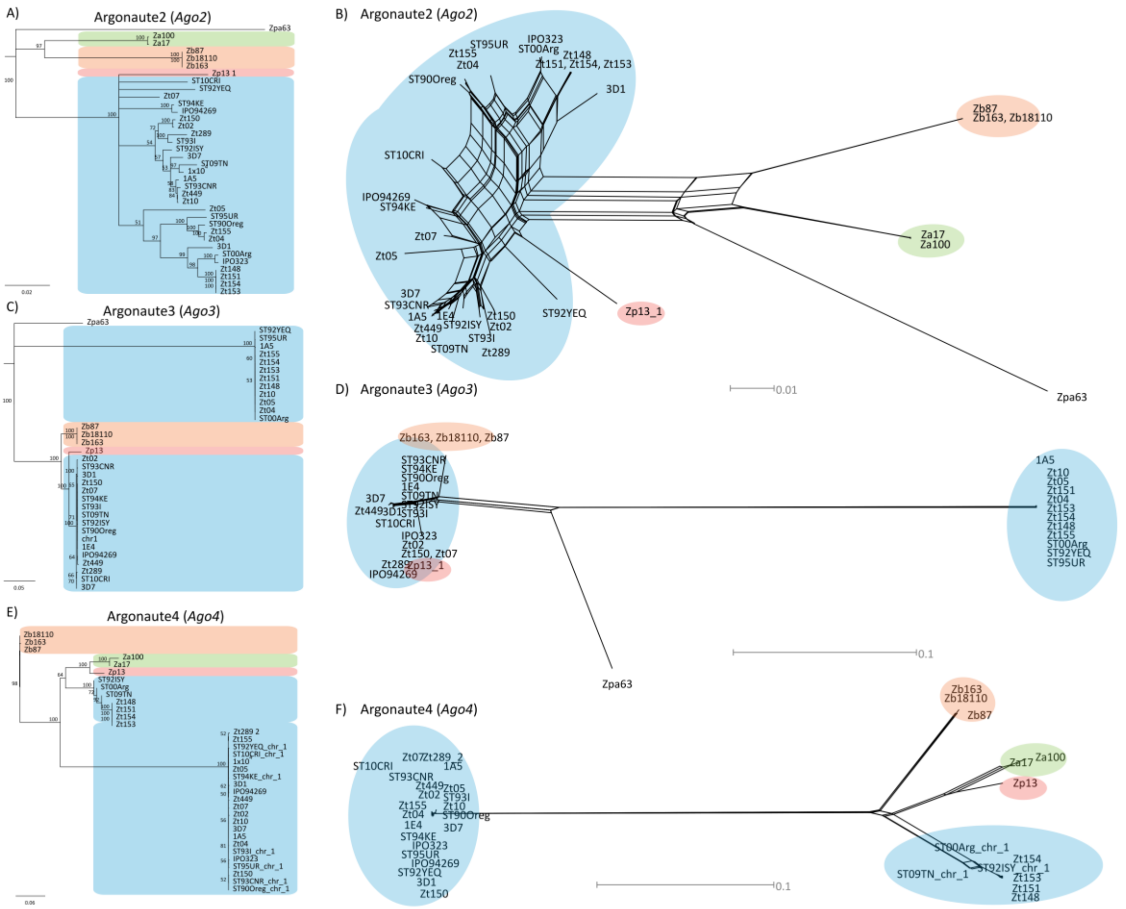

Figure S2. Phylogenetic relationship of *Ago2*, *Ago3*, and *Ago4*. A, C & E) Neighbour-joining tree and B, D & F) phylogenetic network of the respective homologs of in *Z. tritici*, *Z. ardabiliae*, *Z. brevis*, *Z. pseudotritici* and as an outgroup *Z. passerinii* (Zpa63). Support of nodes (% of 1000 bootstraps). C & D). *Ago2* shows one allele group in *Z. tritici* (blue), while *Ago3* and *Ago4* show two allele groups with one clustering with the sister-species of *Z. tritici* (*Z. ardabiliae* (green), *Z. pseudotritici* (red), *Z. brevis* (orange))

A)

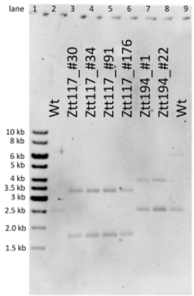

Lane		Strain	Clone	EagI digestion (bp)
2	wt	IPO323 (Zt09)		2513, 6632
3	*Δdcl*	Zt117	#30	1741, 3367
4		Zt117	#34	
5		Zt117	#91	
6		Zt117	#176	
7	*Δdcl::dcl_gfp*	Zt194	#1	2512, 4055
8		Zt194	#22	
9	wt	IPO323 (Zt09)		2513, 6632

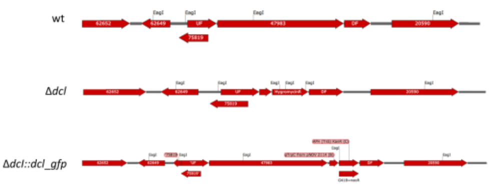

B)

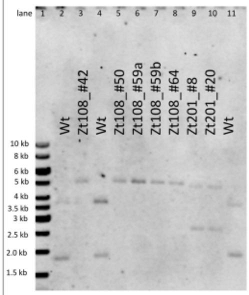

Lane		Strain	Clone	BglI digestion (bp)
2	wt	IPO323 (Zt09)		3659, 1792
3	*Δago1*	Zt108	42	4973
4	*Δago1*	Zt108	45	
5	*Δago1*	Zt108	50	
6	*Δago1*	Zt108	59a	
7	*Δago1*	Zt108	59b	
8	*Δago1*	Zt108	64	
9	*Δago1::ago1_eGFP*	Zt201	8	4832, 2524
10	*Δago1::ago1_eGFP*	Zt201	20	
11	wt	IPO323 (Zt09)		3659, 1792

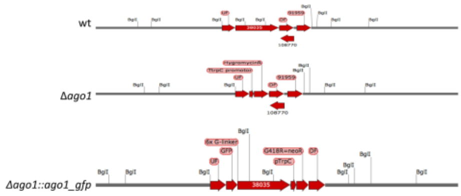

C)

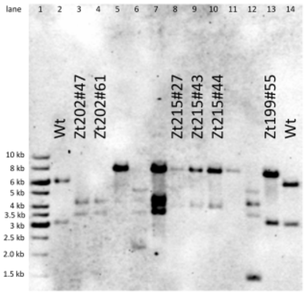

Lane		Strain	Clone	BglII digestion (bp)
2	wt	IPO323 (Zt09)		6068, 3098
3	*Δago2*	Zt202	47	4135,3461
4	*Δago2*	Zt202	61	
5				
6				
7				
8	*Δago2::ago2_gfp*	Zt215	27	7239, 3830
9	*Δago2::ago2_gfp*	Zt215	43	
10	*Δago2::ago2_gfp*	Zt215	44	
11				
12				
13	*Δago2::ago2*	Zt199	55	7238, 3128
14	wt	IPO323 (Zt09)		6068, 3098

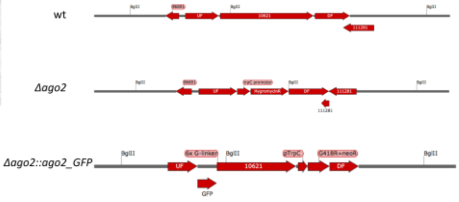

D)

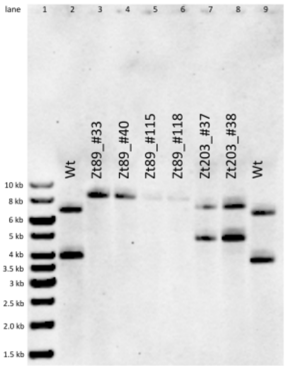

Lane	Genotype	Strain	Clone	SalI digestion (bp)
2	wt	IPO323 (Zt09)		6951, 3964
3	*Δago3*	Zt89	33	8609
4	*Δago3*	Zt89	40	
5	*Δago3*	Zt89	115	
6	*Δago3*	Zt89	118	
7	*Δago3::ago3_gfp*	Zt203	37	7683, 5128
8	*Δago3::ago3_gfp*	Zt203	38	
9	wt	IPO323 (Zt09)		6951, 3964

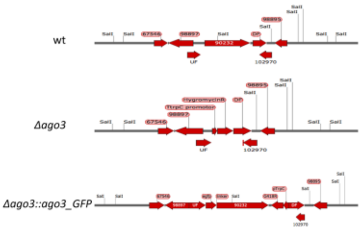

Figure S3. Southern blot confirming the deletion and complementation of *Ago1*, *Ago2*, *Ago3* and *Dcl* in *Z. tritici*. Southern blot results for at least 2 independent clones for each deletion and complementation, table with expected fragment sizes for correct clones and graphical depiction of wildtype locus, locus with deletion and complementation for A) *DcI*, B) *Ago1*, C) *Ago2*, and D) *Ago3*.

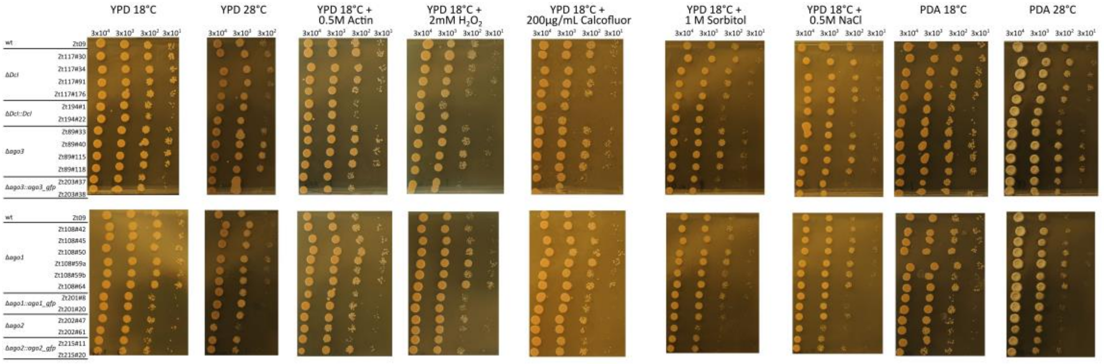

Figure S4. *In vitro* phenotype of wildtype and at least two independent deletion and complementation clones for *DcI, Ago1, Ago2,* and *Ago3*. The indicated number of cells were inoculated for 7 days on YPD or PDA media including the indicated compounds to assess the effect of deletion and complementation on the growth and morphology of *Z. tritici* at higher osmotic stress (NaCl, Sorbitol), increased concentration of reactive oxygen species (H_2O_2), cell wall stressors (Calcofluor) and increased temperature (28°C).

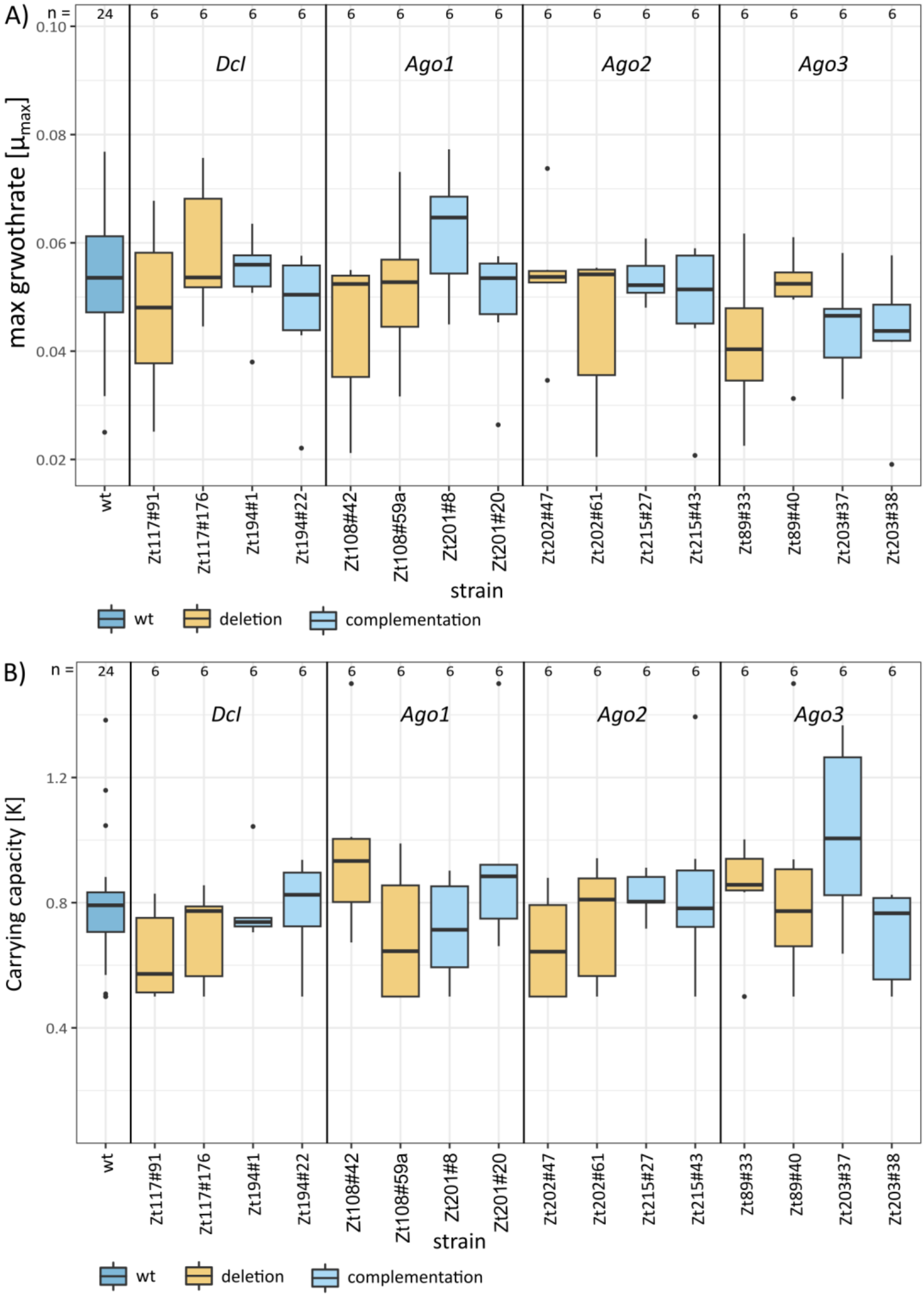

Figure S5. *In vitro* growth phenotype of wildtype and two independent deletion and complementation clones for *Dcl*, *Ago1*, *Ago2*, and *Ago3*. A) Box-whiskers plot of the fitted maximum growth in comparison to the wildtype strain B) Box-whiskers plot of the fitted carrying capacity in comparison to the wildtype strain A) & B) Wilcoxon signed rank test showed no significant differences between the wildtype and any of the deletion or complementation strains.

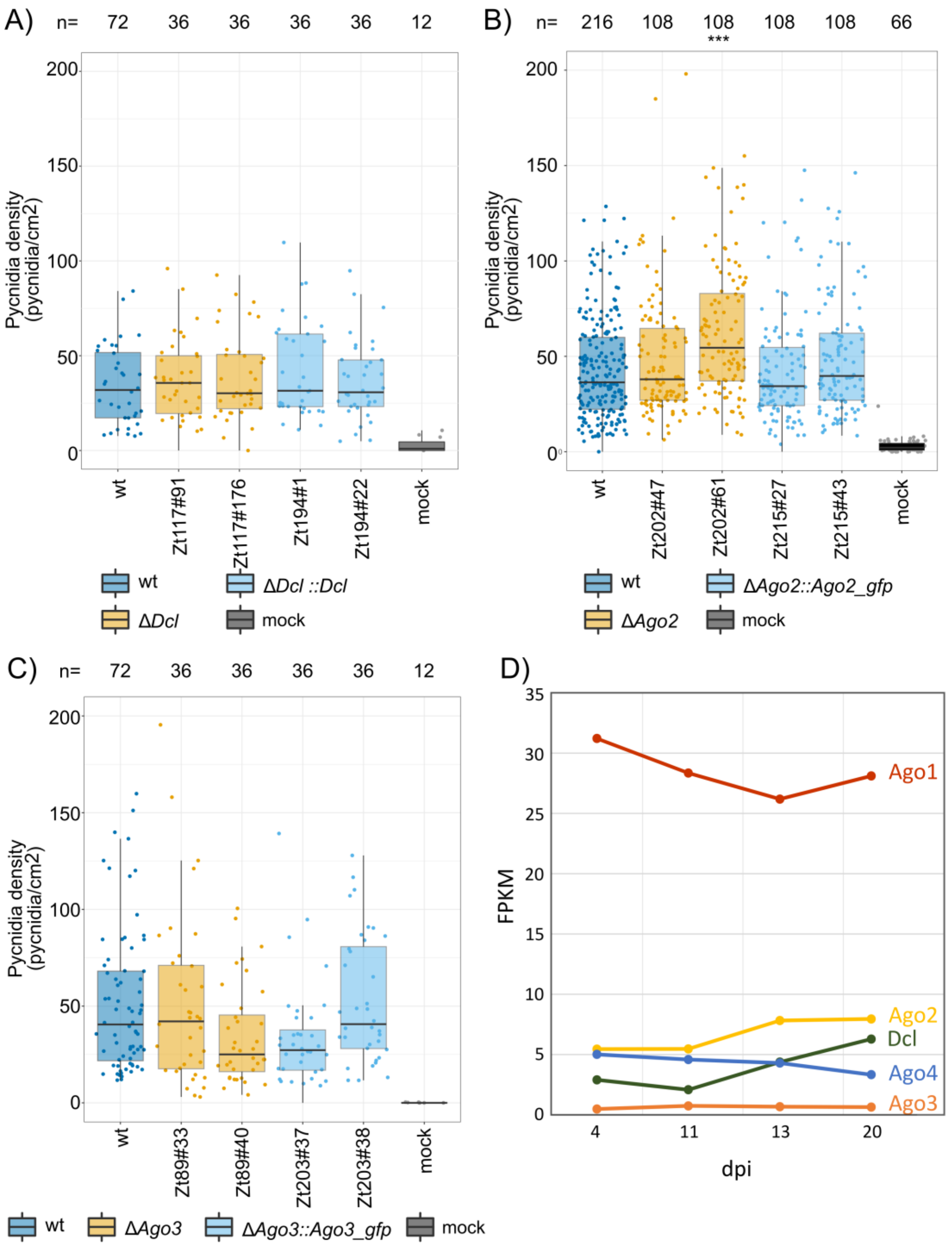

Figure S6. *Ago2*, *Ago3* and *DcI* did not affect the ability of the fungus to propagate *in planta*. (a) Two independent *DcI* deletion strains (Δ*DcI*, orange) and two independent strains where the wt *DcI* allele was complemented in its genomic locus (Δ*DcI*::*DcI*, light blue) showed similar pycnidia densities compared to the wt (dark blue). Data from one experiment is depicted. (b) Two independent Ago2-deletion strains (Δ*Ago2*, orange) varied in their phenotypes *in planta*. Zt202#47 showed similar pycnidia density to the wildtype whereas Zt202#61 showed a significantly higher pycnidia density than the wt. The complementation of the wild type *Ago3* allele in its genomics locus (Δ*Ago2*::*Ago2*-GFP, light blue) did not result in a significant difference in pycnidia density compared to the wt. Pooled data from three independent experiments is depicted. (c) Two independent *Ago3*-deletion strains (Δ*Ago3*, orange) and two independent strains where wt Ago3 allele was complemented in its genomic locus

(Δ*Ago3*::*Ago3*, light blue) showed similar densities in pycnidia compared to the wt IPO323 (dark blue). Data of one experiment is shown. (d) Expression levels of genes of the RNAi machinery in planta at different time points (dpi: days post inoculation, FPKM: Fragments Per Kilobase of transcript per Million mapped reads). Already published RNA-seq data was used [64]. Data depicts mean of two biological replicates. Statistical significance was inferred by an ANOVA on ranked Pycnidia densities using the model pycnidia density ~ strain * (experiments) * (operator), where applicable, with a post hoc Tukey's HSD in a pairwise comparison to the wt. (* = $p<0.05$, ** = $p<0.005$, *** = $p<0.0005$).

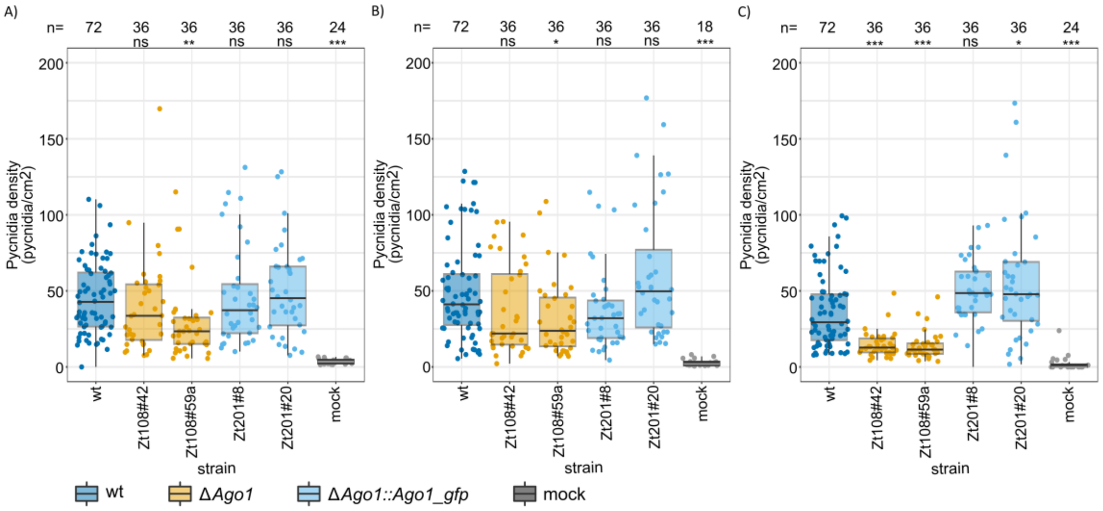

Figure S7. AGO1 affected the ability of the fungus to infect and propagate *in planta* in three independent experiments. Of two independent *Ago1*-deletion strains (Δ*Ago1*, orange) at least one showed a significantly lower density of pycnidia on the leaf surface at 21 dpi (days post inoculation) compared to the IPO323 wildtype (wt, dark blue) in each of the three independent experiments (A) Experiment 1, (B) Experiment 2 and (C) Experiments 3. Complementation of the deletion by the wt *Ago1* allele in its genomic locus (light blue) restored the wt phenotype. Statistical significance was inferred by ANOVA on ranked Pycnidia densities using the model pycnidia density ~ strain * operator with a post hoc Tukey's HSD in a pairwise comparison to the IPO323 wt. Categorized p-values of Tukey's HSD post-hoc test on an ANOVA on ranked pycnidia density are shown (ns: not significant, *: $p<0.05$, **: $p<0.005$, ***: $p<0.0005$).
